# Basic roles of key molecules connected with NMDAR signaling pathway on regulating learning and memory and synaptic plasticity

**DOI:** 10.1186/s40779-016-0095-0

**Published:** 2016-08-31

**Authors:** Hui Wang, Rui-Yun Peng

**Affiliations:** Department of Experimental Pathology, Beijing Institute of Radiation Medicine, Beijing, 100850 China

**Keywords:** N-methyl-D-aspartic acid receptors, Long-term potentiation, Synaptic plasticity, Learning and memory

## Abstract

With key roles in essential brain functions ranging from the long-term potentiation (LTP) to synaptic plasticity, the N-methyl-D-aspartic acid receptor (NMDAR) can be considered as one of the fundamental glutamate receptors in the central nervous system. The role of NMDA R was first identified in synaptic plasticity and has been extensively studied. Some molecules, such as Ca^2+^, postsynaptic density 95 (PSD-95), calcium/calmodulin-dependent protein kinase II (CaMK II), protein kinase A (PKA), mitogen-activated protein kinase (MAPK) and cyclic adenosine monophosphate (cAMP) responsive element binding protein (CREB), are of special importance in learning and memory. This review mainly focused on the new research of key molecules connected with learning and memory, which played important roles in the NMDAR signaling pathway.

## Background

**Fig. 1:**
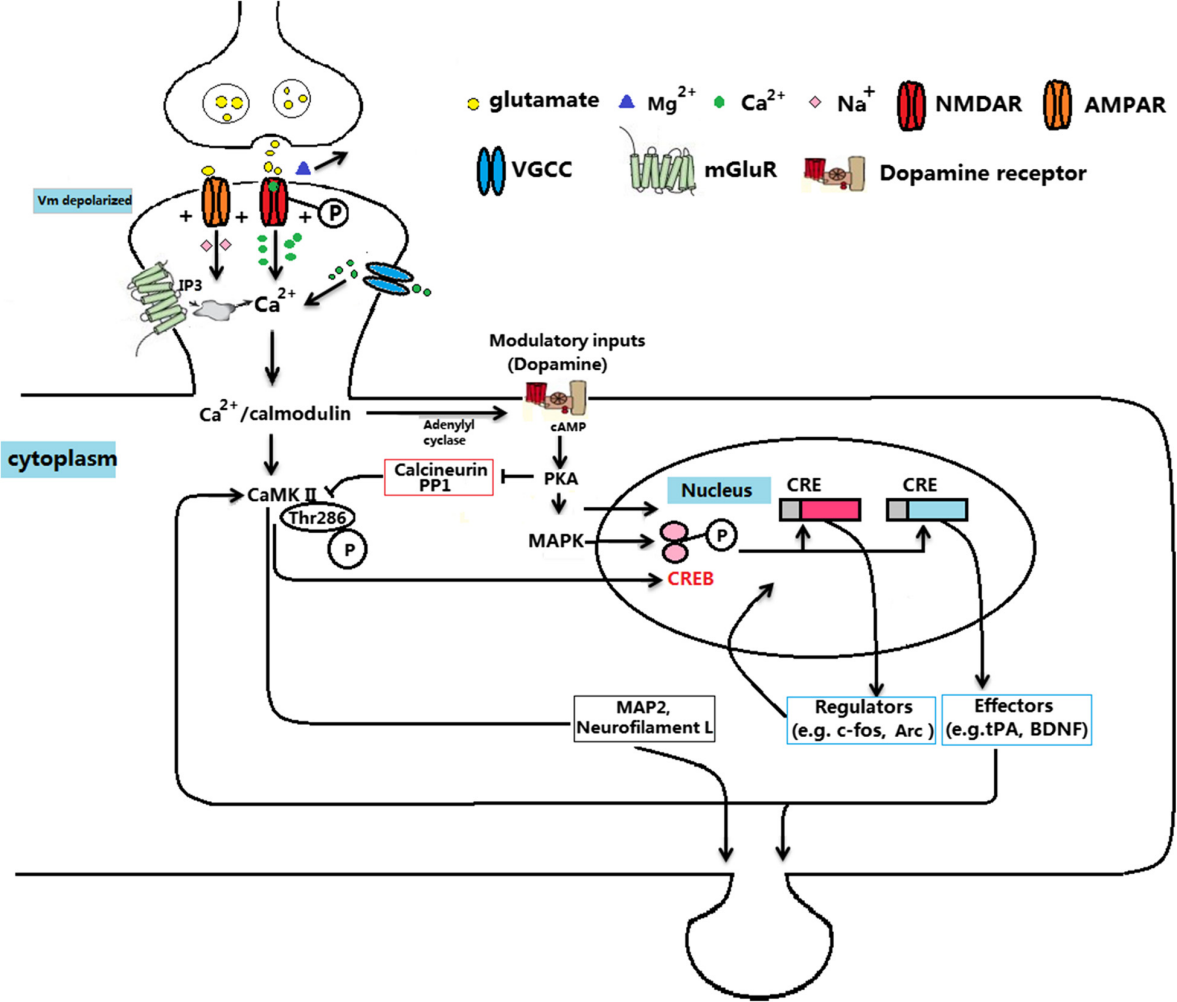
The regulation of molecules in the NMDAR signaling pathway on learning and memory. The release of presynaptic glutamate can activate postsynaptic NMDAR, leading to the removal of magnesium ions and the influx of calcium. The calcium could bind to the calmodulin to activate the calmodulin-dependent PKA and PKC, which are the basis for the activation of MAPK. Furthermore, the activated MAPK can be transported into the nucleus and activate CREB, resulting the expression of downstream genes, such as tPA and BDNF, among others. Finally, the new synthetic substances can cause the growth of original synapses and the formation of new synapses, which are the basis for synaptic plasticity and learning and memory

Glutamate, one of the most common neurotransmitters in the central nervous system, has been found to regulate various memory-connected activities. Glutamate receptors, as the most important excitatory amino acid receptors in the mammalian central nervous system, have been confirmed to be associated with the formation of synapses, the release and transport of neurotransmitters, synaptic plasticity and learning and memory [1]. N-methyl-D-aspartic acid receptor (NMDAR) is an important glutamate receptor, which has been proven to be closely related to long-term potentiation (LTP), learning and memory, the development of neural plasticity, hypoxic-ischemic injuries and some neurodegenerative diseases [2]. The role of NMDAR in regulating learning and memory has been confirmed by different experimental methods, such as gene knockout or overexpression, electrophysiological techniques, agonists, and antagonists [3–5]. Some key molecules of NMDAR signaling pathway in postsynaptic neurons play important roles in regulating learning and memory, which can be summarized as the following processes. Firstly, the release of presynaptic glutamate can activate the postsynaptic NMDAR, leading to the removal of magnesium ions and the influx of calcium. Then, the calcium could bind to the calmodulin to activate the calmodulin-dependent protein kinase and protein kinases A and C, which are the basis for activating mitogen-activated protein kinase (MAPK). Furthermore, the activated MAPK can be transported to the nucleus and activate cyclic adenosine monophosphate (cAMP) responsive element binding protein (CREB), resulting in the expression of downstream genes, such as tissue-type plasminogen activator (tPA) and brain-derived neurotrophic factor (BDNF), among others. Finally, the new synthetic substances can cause the growth of original synapses and the formation of new synapses (Fig. 1). The aim of this review is to discuss the important processes connected with NMDAR dependent learning and memory.

### Activation of NMDARs and the influx of calcium

NMDARs, voltage- and ligand-gated channels, are found throughout the central nervous system and contribute to synaptic plasticity and intracellular Ca^2+^ transients. NMDARs can be activated by the binding of presynaptic glutamate as well as through postsynaptic depolarization. At the rest potential, when the presynaptic membrane is activated, many neurotransmitters are released into the synaptic cleft, including glutamate. The released glutamate can bind to both α-postsynaptic amino-3-hydroxy-5-methyl-4-isoxazole propionic acid receptor (AMPAR) and NMDAR. Under this condition, barely any ions can flow through the NMDARs because of the negative resting potential and the Mg^2+^ blocking site. However, the Na^+^ and K^+^ can pass through AMPARs, resulting in the depolarization of the postsynaptic membrane. Furthermore, a large depolarized current would cause the Mg^2+^ to become unbound from NMDAR, leading to the open state of the NMDAR channel and the subsequent influx of Ca^2+^, which is one of the triggering factors for the LTP induction and maintenance [6]. Sanna et al. [7] found that the transient use of glutamate or NMDA at the dendrites of pyramidal cells could increase the intracellular Ca^2+^ concentration, however this reaction can be inhibited by the NMDAR antagonist AP-5 or extracellular Mg^2+^. The phosphorylation status of NMDAR is an important factor that reflects the activation status of NMDAR and determines the permeability of calcium ions. In other words, their permeability is enhanced by phosphorylation, whereas dephosphorylation decreases calcium permeability [8, 9].

In addition to the NMDAR channel, the voltage-gated calcium channel (VGCC), metabotropic glutamate receptor (mGluR) and cytosolic calcium stores, including the endoplasmic reticulum, are other important ways for the increase of intracellular calcium ions [10, 11]. The increased Ca^2+^ in postsynaptic neuron contributes to the rearrangement of cell scaffolding proteins, the increase of postsynaptic area and the decrease of resistance during synaptic transmission, leading to the formation of LTP [12, 13]. Moreover, the calcium, as an important intracellular second messenger, can activate several important protein kinases, such as protein kinase A (PKA), calcium/calmodulin-dependent protein kinase II (CaMK II), and MAPK, among others, resulting in the start of a downstream signal pathway.

### Bridging roles of PSD-95 in postsynaptic plasticity

Postsynaptic density, located at the cytoplasmic membrane, is a large semicircular area of high electronic density. The main components of postsynaptic density are types of receptors, including CaMK II and postsynaptic density 95 (PSD-95), among others. One study found that the CaMK II and PSD-95 were mainly bound to the NR2B subunits [14]. Another study found that hippocampal synaptic plasticity was related to the competitive binding of the C-terminus of NR2A to either CaMK II or PSD-95 [15].

PSD-95, an important scaffold protein in the postsynaptic membrane, serves as a bridge between NMDARs and downstream signal molecules and plays roles in the maturation of neurons and the development of synapses. In addition, PSD-95 binds various regulatory proteins, including Src, Pyk2, SynGAP, and nNOS and may recruit signaling proteins to NMDARs [16]. Studies have found that the overexpression of PSD-95 in hippocampal neurons can drive the maturation of glutamatergic synapses as well as enhance postsynaptic clustering, the activity of glutamate receptors and the maturation of presynaptic terminal, thus indicating its roles in synapse stabilization and plasticity [17, 18]. In PSDs, NR2A and NR2B subunits directly interact with PSD-95 and other members of the membrane-associated guanylate kinase family. Studies found that the PSD-95-like membrane-associated guanylate kinases (PSD-MAGUKs) specifically played roles in clustering signaling molecules near NMDAR. The ligand binding-deficient PSD-95 cDNA knockin mice presented with markedly abnormal anxiety-like behavior, impaired spatial reference and working memory, and impaired remote memory and pattern separation in fear conditioning tests [19]. TrkB, a BDNF receptor, regulates the postsynaptic localization of PSD-95 through the following three downstream pathways: phosphatidylinositol 3-kinase (PI3K), phospholipase Cγ (PLCγ) and MAPK/extracellular signal-regulated kinases (MAPK/ERK) [20].

### Autophosphorylation of CaMK II and its role in learning and memory

CaMK II, a serine/threonine kinase, is activated during the induction of LTP through the calcium ion influx through NMDAR [21]. CaMK II has more than 30 types of subtypes and multiple substrates. CaMK II is likely a mediator of primary importance in linking transient calcium signals to neuronal plasticity. When NMDAR is activated, calcium ions are transported through the receptor channel and then bind to calmodulin (CaM). The Ca^2+^/CaM complex can then bind to the contiguous sequences and consequently displace the Ca^2+^ from the CaM catalytic domain, leading the autophosphorylation of CaMK II at Thr286 [22]. The activated CaMK II can then move to the PSD and subsequently induce LTP. Studies found that the synaptic strength was stored stably through the combined actions of the CaMK II/NMDAR complex and N-cadherin dimers, which have redundant storage that could provide informational stability in a manner analogous to base-pairing in DNA [23]. In the maintenance of LTP, the CaMK II can bind to a number of postsynaptic density proteins, such as α-actinin, PSD-95, synaptic adhesion molecule, and densin-180. The activated CaMK II can also cause the activation of microtubule-associated protein 2 (MAP2) and neurofilament L, indicating the close relationship of CaMK II and structural synaptic plasticity [24]. The synaptic scaffolding protein CaM associated serine kinase (CASK) is also important for learning and memory, as mutations in CASK result in intellectual disability and neurological defects in humans. Previous research has found that the expression of human CASK in *Drosophila* rescued the effect of CASK deletion on the activity state of CaMK II, suggesting that human CASK may also regulate CaMK II autophosphorylation in neuronal growth, calcium signaling, and learning [25].

To test the role of CaMK II autophosphorylation in synaptic plasticity, research studies were conducted with transgenic mice that express CaMK II-Asp-286 instead of CaMK II-Thr-286 [26]. Although the calcium-independent activity increased two times, the hippocampal LTP induction at CA1 region was inhibited and the spatial memory was impaired when the mice were subjected to a Morris water maze [27]. Above all, CaMK II plays a key role during LTP induction by enhancing alternations in synaptic efficiency [28].

### The production of cAMP and activation of PKA and MAPK

The increase of calcium ions in postsynaptic neurons can activate adenylyl cyclase, leading to the expression of cAMP and the activation of PKA. The increased cAMP could cause MAPK to be transported to the nucleus.

PKA plays a role in the cytoplasm, which can phosphorylate different substrates, for example, GluR1 of AMPAR, which can counteract protein phosphatase 1 (PP1), facilitate LTP induction and impact learning and memory [29]. Experiments showed that the incidence of LTP induction could be decreased by the antagonist of PKA, indicating the PKA was a key molecule for LTP [30]. The activation of PKA or the inhibition of PP1 can increase the NMDAR dependent postsynaptic potentials [31]. Researchers generated transgenic mice that express R(AB), an inhibitory form of the regulatory subunit of PKA, finding deficits in long-term memory and confirming the critical role of PKA in the consolidation of long-term memory [32].

There are four subtypes of MAPK, namely extracellular signal-regulated protein kinase (ERK), p38 mitogen-activated protein kinase (p38 MAPK), c-jun N-terminal kinase (JNK, also known as stress-activated MAPK) and ERK5. After the phosphorylation of MAPK, MAPK can enter the nucleus and phosphorylate nuclear transcription factors, leading to the expression of downstream target genes and the synthesis of new proteins [33, 34]. MAPK is indispensable to generating hippocampal LTP. The extracellular signal-regulated kinase 1/2 (ERK1/2) is a best characterized subclass of MAPKs; its phosphorylation was resistant to the inhibition of protein kinase C, p38 MAPK, cyclin-dependent kinase 5, and receptor tyrosine kinase (epidermal growth factor receptors) or non-receptor tyrosine kinases (including Src) by their selective inhibitors [35]. One research study reported that PD 098059, a selective inhibitor of the MAPK cascade, blocked MAPK activation in response to the direct stimulation of NMDAR as well as to LTP-inducing stimuli, which provided evidence of its role in the MAPK cascade, specifically in the activity-dependent modification of synaptic connections between neurons in the adult mammalian nervous system [36].

In addition to protein kinase, protein phosphatase also regulates LTP and memory. However, unlike the PKA and MAPK cascades, the protein phosphatase 1 and calcineurin are negative regulation factors for LTP formation. Researchers found that the physiological importance of PP1 and calcineurin constrained LTP and memory [37–39].

### The phosphorylation of CREB and its regulatory roles

CREB, one of the earliest confirmed and phosphorylation regulated transcription factors, is expressed in nearly all cells of the brain and is localized in the nucleus. CREB regulates the gene transcription of cAMP at the promoter region and a large number of downstream target genes induced by a variety of signaling molecules. The phosphorylation of CREB affects the expression of many neuronal genes and proteins, regulating the entire neural network [40, 41]. Moreover, CREB is also involved in sperm formation and the regulation of circadian rhythms [41].

Different studies have confirmed the important roles of CREB phosphorylation mainly in long-term learning and memory, involving procedures on *Aplysia*, flies, and rodents [42–44]. In organotypic hippocampal slices, CREB phosphorylation continued to increase for 4 h during LTP maintenance, supporting the hypothesis that CREB plays an important role during the late phases of LTP [45]. Matsushita et al. [46] found that the PKA inhibitory peptide blocked both CREB phosphorylation and long-lasting LTP induction but not early LTP. Acute expression of active CREB caused an enhancement of both NMDA R-mediated synaptic responses and LTP, which was also accompanied by electrophysiological and morphological changes consistent with the generation of “silent synapses” [47]. The phosphorylation levels of ERK and CREB and the mRNA levels of CREB target genes (c-fos and Nr4a1) were significantly upregulated in the cerebral cortices of NR2B transgenic mice compared to their wild-type littermates, indicating that NR2B overexpression leads to the enhancement of specific protein phosphorylation in the brain [48]. CREB phosphorylation is a fundamental process of signaling cascades, which is connected with the subsequent protein synthesis and the formation of new dendrites. Additionally, the activation of CREB is an important regulator of BDNF-induced gene expression and plays a central role in mediating neurotrophin response in neurons [49].

### The immediate early gene expression and synaptic protein synthesis

The phosphorylation of CREB, the rapid activation of immediate early gene (IEG) and protein synthesis are three factors that can induce LTP. Once the CREB phosphorylation is inhibited, IEG expression and protein synthesis are unable to proceed [50, 51]. IEG, as a transcription factor for the induction of late response genes, can bind to the nuclear DNA regulatory region through its products. The protein products of late response genes mainly include structural proteins, enzymes, ion channels, and neurotransmitters that participate in the regeneration of neurons and synaptic plasticity [52].

IEG includes zif268, c-fos, c-jun, c-myc and activity-regulated cytoskeleton-associated protein (Arc), which can link the short term signals to the long term changes. Among IEGs, the c-fos and Arc play key roles for the maintenance of LTP. Research has found that the increase in zif/268 expression was more highly correlated with LTP duration than with the magnitude of initial LTP [53]. In addition, the mRNA and protein expressions of c-fos related genes, including c-jun, junB, and junD, increased when the LTP was induced [54–56]. zif268 and c-Fos displayed markedly different patterns of hippocampal and prefrontal expression, with zif268 being more closely linked to spatial learning [57]. The Arc mRNA can be transported into the dendrites and generate cytoskeleton associated proteins in a few minutes after being highly stimulated, regulating the structural synaptic plasticity [58].

Long-term memory was considered to be mediated by protein synthesis-dependent, late-phase LTP. Two secreted proteins, tPA and BDNF, were thought to play important roles in the long-term memory. tPA, a major product of the IEG, is a serine protein kinase in mammals, which can decompose the plasminogen to plasmin. The tPA knockout mice appeared to have a selective impairment of hippocampal LTP induction, whereas the tPA-overexpression mice exhibited enhanced LTP. Moreover, tPA also participates in anxiety-related behavior, spatial memory and the induction of cerebellar motor memory [59]. Pang et al. [60] reported that tPA, by activating the extracellular protease plasmin, converted the precursor proBDNF to the mature BDNF (mBDNF) and that such conversion was critical for L-LTP expression in the hippocampus.

### Perspectives

The regulation of NMDAR in learning and memory has been well-recognized by neurobiologists. The neural basis of learning and memory is synaptic plasticity. LTP is an indispensable model for studying the mechanism of NMDAR-dependent learning and memory. So far, the molecules involved in the NMDAR signaling pathway, including calcium ions, PSD-95, CaMK II, PKA, MAPK, CREB, and IEG, have been confirmed to be closely connected with LTP and synaptic plasticity. However, the relationship between NMDAR and other receptors, such as dopamine receptors, GABA receptors, and mGluRs, among others, remains to be studied. Other factors, including aging, hypoxia, ischemia, lead poisoning, and microwave radiation, among other factors, on memory and their mechanisms have not been fully elucidated. We believe the application of new technical methods will better elucidate the mechanism of learning and memory.

## Conclusions

In summary, NMDAR, as an important glutamate receptor, has been proven to be closely related to LTP, learning and memory, and the development of neural plasticity. Key molecules connected with the NMDAR signaling pathway, such as calcium ions, PSD-95, CaMK II, PKA, MAPK, CREB and IEG, participate in the formation of learning and memory and synaptic plasticity. The main process includes the activation of NMDAR and the influx of calcium, the bridging roles of PSD-95 in postsynaptic plasticity, the autophosphorylation of CaMK II and its role in learning and memory, the production of cAMP and the activation of PKA and MAPK, the phosphorylation of CREB and its regulatory roles and IEG expression and synaptic protein synthesis. The competition of the above process requires the precise regulation of every molecule in the NMDAR connected signaling pathway. Therefore, the research on the key molecules connected with the NMDAR signaling pathway plays an irreplaceable role in revealing the mechanism of learning and memory and synaptic plasticity.

## Abbreviations

AMPAR: a-postsynaptic amino-3-hydroxy-5-methyl-4-isoxazole propionic acid receptor
Arc: Activity-regulated cytoskeleton-associated protein
BDNF: Brain-derived neurotrophic factor
cAMP: Cyclic adenosine monophosphate
CaMK II: Calcium/calmodulin-dependent protein kinase II
CaM: Calmodulin
CASK: CaM associated serine kinase
CREB: cAMP responsive element binding protein
ERK: Extracellular signal-regulated protein kinase
IEG: Immediate early gene
LTP: Long-term potentiation
MAPK: Mitogen-activated protein kinase
mGluR: Metabotropic glutamate receptor
NMDAR: N-methyl-D-aspartic acid receptor
PKA: Protein kinase A
PSD-95: Postynaptic density 95
PP1: Protein phosphatase 1
tPA: Tissue plasminogen activator
VGCC: Voltage-gated calcium channel
